# The Abundance of Short Proteins in the Mammalian Proteome

**DOI:** 10.1371/journal.pgen.0020052

**Published:** 2006-04-28

**Authors:** Martin C Frith, Alistair R Forrest, Ehsan Nourbakhsh, Ken C Pang, Chikatoshi Kai, Jun Kawai, Piero Carninci, Yoshihide Hayashizaki, Timothy L Bailey, Sean M Grimmond

**Affiliations:** 1 Genome Exploration Research Group (Genome Network Project Core Group), RIKEN Genomic Sciences Center, RIKEN Yokohama Institute, Yokohama, Japan; 2 Institute for Molecular Bioscience, University of Queensland, Brisbane, Queensland, Australia; 3 Ludwig Institute for Cancer Research, Austin and Repatriation Medical Centre, Heidelberg, Victoria, Australia; 4 Genome Science Laboratory, Discovery Research Institute, RIKEN Wako Institute, Wako, Japan; The Jackson Laboratory, US; MRC-Harwell, UK; NHGRI-NIH, US; Lawrence Livermore National Laboratory, US; The Jackson Laboratory, US

## Abstract

Short proteins play key roles in cell signalling and other processes, but their abundance in the mammalian proteome is unknown. Current catalogues of mammalian proteins exhibit an artefactual discontinuity at a length of 100 aa, so that protein abundance peaks just above this length and falls off sharply below it. To clarify the abundance of short proteins, we identify proteins in the FANTOM collection of mouse cDNAs by analysing synonymous and non-synonymous substitutions with the computer program CRITICA. This analysis confirms that there is no real discontinuity at length 100. Roughly 10% of mouse proteins are shorter than 100 aa, although the majority of these are variants of proteins longer than 100 aa. We identify many novel short proteins, including a “dark matter” subset containing ones that lack detectable homology to other known proteins. Translation assays confirm that some of these novel proteins can be translated and localised to the secretory pathway.

## Synopsis

Living things work by the actions of proteins and other molecules that are encoded in their genomes. Genome projects aim to construct a “parts list” of life by cataloguing all of these molecules. However, some molecules are harder to identify than others. One difficult category is short proteins. This is because protein-coding nucleotide sequences have statistical features that, for large proteins, are very pronounced and readily distinguishable from non-coding sequence, but for small proteins are not. Thus, to avoid erroneous predictions, many genome projects have employed an artificial length threshold of 100 amino acids. Hence, short proteins are underrepresented in protein catalogues, although they are known to play important roles in immunity, cell signalling, and metabolism. This study clarifies the abundance of short proteins by exploiting two advantageous resources. The first is the large FANTOM collection of mouse transcript sequences: it is much easier to identify proteins in mature transcripts than in raw genome sequence. The second is a method to identify protein-coding sequences by examining how they differ between organisms. The redundancy of the genetic code implies that these differences will follow distinctive patterns, and this approach has the statistical power to break the 100-amino-acid barrier and reliably identify short proteins.

## Introduction

Large-scale cDNA annotation has often been performed under the assumption that protein-coding transcripts encode for peptides of 100 aa or longer. Although this is a quite useful generalisation, the existence of mRNA coding for short proteins needs to be addressed. Short proteins are important mediators of biological processes that include (1) regulation of innate immunity (via more than a dozen members of the small inducible cytokine families CCL and CXCL), (2) protection against pathogens (via more than two dozen of the xenobiotic defensin and defensin-related cryptidin factors), (3) cell communication and homeostasis as ligands and hormones (e.g., Apln, Gnrh1, and Ppy), (4) signal transduction (e.g., the Pki protein kinase inhibitor and Gng guanine nucleotide binding protein–gamma families), and, finally, (5) metabolism (e.g., playing key roles in mitochondrial electron transport, cytochrome C subunit, and co-enzyme metabolism). Because of their small size, these peptides are likely to possess distinct roles and properties (e.g., ontological review of peptides less than 100 aa in Swiss-Prot reveals that almost a third reside in the extracellular compartment). Thus, short proteins less than 100 aa carry out many essential functions, and are particularly associated with cell–cell communication, which is the basis of multicellular life. However, current open reading frame (ORF) predictions and annotation projects are missing them.

**Figure 1:**
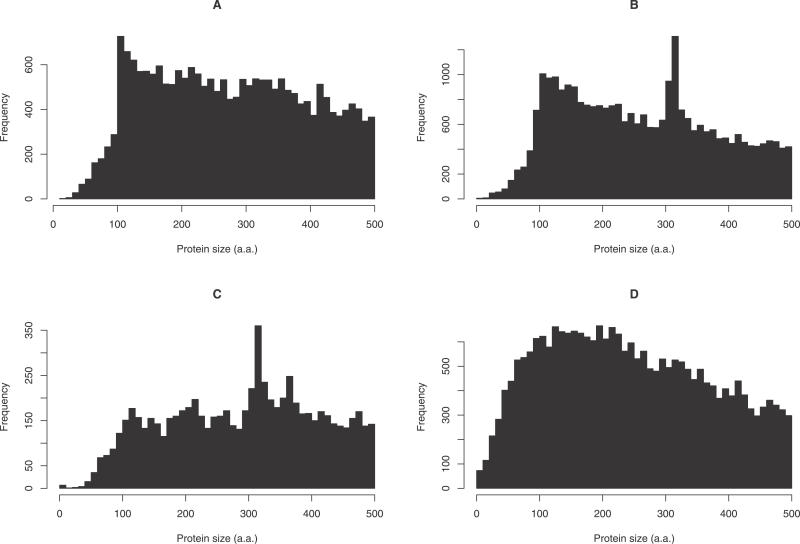
Size Distributions of Mammalian Proteins (A) For 29,991 full-length mouse proteins from the FANTOM annotations. (B) For 40,865 mouse proteins from the IPI database. (C) For 11,679 human proteins from Swiss-Prot. (D) For 31,035 mouse proteins predicted in the FANTOM cDNAs using CRITICA.

The FANTOM consortium sequenced a large set of full-length mouse cDNAs and manually annotated their protein-coding capacity [1,2]. Only 987 out of 29,991 FANTOM proteins (3.3%) were annotated as shorter than 100 aa. When one plots the peptide length distribution for this set, it is clear that there is a sudden drop in the number of cDNAs encoding peptides less than 100 aa (Figure 1A). This curious length distribution is not too surprising since ORF length greater than 100 codons was one of the criteria used to annotate proteins [1] (N Maeda et al. in preparation). Such distributions are also apparent in other catalogues, including the mouse IPI and human Swiss-Prot sets (Figure 1B and 1C).

The IPI set is a comprehensive database of known and predicted mouse proteins obtained by integrating several source databases [3]. The peptide length distribution exhibits a spike at 300–310 aa, corresponding to the large olfactory receptor family, which is missing from the FANTOM set because olfactory tissues were not sampled deeply (Figure 1). Only 1,835 out of 40,865 IPI proteins (4.5%) are shorter than 100 aa. Even this small number may be contaminated with fragments of longer proteins, since proteins predicted from genome sequence are often partial [4].

In contrast, Swiss-Prot is a manually curated protein database, which emphasises accuracy at the expense of completeness [5]. Unlike the other datasets, the human Swiss-Prot proteins do not exhibit a rise in abundance from 500 aa down to 100 aa, but the sharp decline below 100 aa is still present (Figure 1C). Splice variants are merged in the Swiss-Prot set whereas they are separate in the FANTOM and IPI sets; however, a sharp drop-off in the abundance of short proteins is apparent in either case. Only 405 out of 11,679 human Swiss-Prot proteins (3.5%) are shorter than 100 aa. Even supposing that Swiss-Prot contains no errors, it is easy to imagine how a biased length distribution could arise from ascertainment bias in the protein reports available for inclusion into Swiss-Prot.

This short-length discontinuity has been noted previously for yeast, bacterial, and mouse proteins [6,7]. Das et al. [6] plotted length distributions for yeast proteins with and without “clear” homologues, and observed a striking peak at 100–110 aa in the latter but not the former distribution, suggesting over-prediction of proteins just over 100 aa. However, the authors did not comment on another feature of their plots: a marked drop in the number of proteins with clear homologues below 100 aa. This suggests under-prediction of proteins less than 100 aa.

Several previous studies have attempted to clarify the abundance of short proteins encoded in microbial genomes. Ochman measured ratios of synonymous to non-synonymous substitutions between homologous ORFs in pairs of bacteria [8]. Protein-coding ORFs have an excess of synonymous versus non-synonymous substitutions, if the protein sequence is conserved between the species, whereas non-coding ORFs have a ratio close to one. Choosing a *K*_a_/*K*_s_ threshold is not straightforward, however, and there may be some translated, functional proteins with little or no conservation of the protein sequence, such as leader peptides [9]. Kellis et al. identified protein-coding ORFs in yeast by measuring the proportion of the ORF over which the reading frame is conserved in alignments with other yeast genomes [10]. Harrison et al. attacked this problem by comparing the “polyORFome”—all possible ORFs in 65 microbial genomes—to itself at the protein level to identify conserved proteins [11]. This kind of approach risks spurious matches of highly conserved non-coding sequences, or, if the homology criteria are made very strict to avoid this problem, proteins with weak conservation or limited phyletic distribution will be missed. For mammals, this dilemma is enhanced by the existence of non-coding sequences that are more conserved than any protein [12]. Overall, synonymous/non-synonymous analysis seems like a sharper tool for discriminating protein-coding ORFs.

These studies have not been extended to multicellular eukaryotes, partly because gene finding is much harder in these large and intron-rich genomes, and perhaps partly because of an erroneous assumption that protein identification from extensive cDNA data is trivial. cDNA sequences certainly have advantages over genomes: introns and most pseudogenes are absent, the search space is much reduced, and, classically, each mRNA should encode precisely one protein. The main difficulties are that there may actually be many non-coding RNAs [1,13], which are difficult to distinguish from mRNAs encoding short proteins, and that cDNAs may suffer from various experimental artefacts such as truncation and intron retention.

To clarify the contribution of small proteins to the mammalian proteome, we identify proteins in the FANTOM collection of 102,801 mouse cDNAs. This is the largest available cDNA set, collected from many tissues and developmental stages, and, importantly, it was constructed without explicit biases towards long or short proteins. This property is not always true of other cDNA collections; for instance, the Kazusa Human cDNA Project targets large proteins [14]. For this reason we do not use all public cDNAs. A final advantage of FANTOM is that the cDNAs and the public genome sequence come from the same inbred mouse strain (C57BL/6J), which facilitates various analyses such as checking for sequencing errors.

To identify proteins with minimal length bias, and discriminate protein-coding from non-coding RNA, we use the CRITICA (Coding Region Identification Tool Invoking Comparative Analysis) suite of programs [15]. CRITICA was designed to identify protein-coding ORFs in bacterial genomes by combining comparative analysis, similar to Ochman's synonymous/non-synonymous measurements, with a statistical analysis of coding sequences (CDSs). In its first step, CRITICA scans pair-wise alignments of homologous nucleotide sequences, and identifies regions whose translation has a greater amino acid identity than expected for the observed percentage nucleotide identity. Next, the program tallies hexanucleotide frequencies in predicted coding and non-coding frames, and combines this information with the comparative data to re-predict the coding regions more accurately. This hexanucleotide counting and re-prediction is iterated several times. CRITICA calculates *p*-values indicating the probability that a region with this degree of coding evidence would arise by chance, and outputs predictions with *p*-values less than 0.0001. By utilising powerful comparative and hexamer evidence in a statistically rigorous fashion, CRITICA is a particularly promising approach for accurately identifying proteins below the 100-aa barrier, and we provide evidence below that it does so successfully. This approach eliminates the biases of typical protein identification pipelines because it does not employ a length threshold, and it does not rely on comparison to pre-existing protein collections that may have length biases.

## Results

The CRITICA pipeline was used to identify proteins encoded in the 102,801 FANTOM cDNA sequences. Since CRITICA was designed for bacterial DNA, it may predict proteins in reverse strands or more than one protein per sequence. Reverse-strand predictions were removed: they occur when a transcript is antisense to a protein-coding exon. There were 9,625 cDNAs with multiple forward-strand predictions: these often have unspliced introns or frameshift sequencing errors, and were discarded. A further 3,344 predictions lacked stop codons, so that the coding region runs off the end of the cDNA (putative 3′ truncated cDNAs); these were also removed, leaving 49,378 predictions. These initial filtering steps are summarised in Table 1.

**Table 1:**
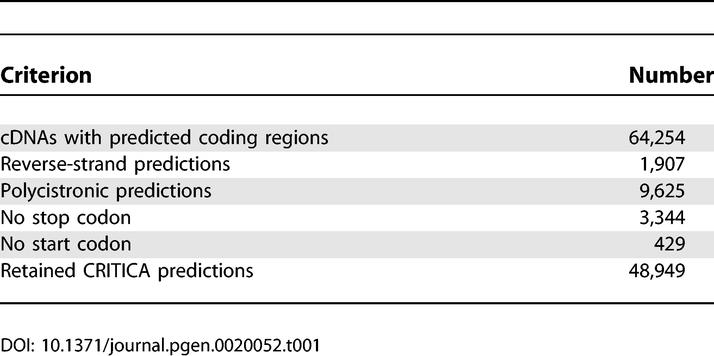
Initial Filtering of CRITICA CDS Predictions for the FANTOM cDNAs

CRITICA does not seem to predict start codons very reliably. In particular, the protein-coding region is predicted to begin before the start of the cDNA (putative 5′ truncated cDNAs) in 22,112 cases (45%), whereas 12,147 of these have internal start codons according to each of three other protein identification methods employed by the FANTOM consortium (DECODER, rsCDS, and mTRANS). Examination of CRITICA's intermediate .crit file revealed that 14,111 of these cDNAs had alternative non-truncated predictions with *p*-values less than 10-fold higher than the truncated predictions. So CRITICA cannot confidently discriminate alternative start locations, which is not too surprising since it lacks a component for analysing eukaryotic start signals. (It has a component for predicting bacterial Shine-Dalgarno sequences, which we turned off.) Therefore, we employed an extra rule to get the final protein predictions: the most upstream possible ATG was used as the start codon. The ribosome scanning model suggests that the upstream-most ATG will usually be correct [16]. In addition, since stop codons occur three times as often as start codons, there will usually be an intervening stop between the true start codon and any upstream ATG. Nevertheless, we expect this procedure to cause a slight underestimate in the abundance of short proteins. For 429 cDNAs, no ATG could be found, and these were discarded, leaving protein predictions from 48,949 cDNAs (Datasets S1 and S2).

### Redundancy

The FANTOM collection includes transcripts from overlapping genomic regions. In some cases the predicted protein-coding regions in two transcripts derive from identical genomic exons, so that the 48,949 cDNAs encode 31,035 genomically distinct proteins. (Of these, 187 cDNAs were not uniquely mapped to the genome and are excluded from the protein count.) In other cases the predicted protein-coding regions derive from partially overlapping genomic exons, owing to alternate usage of splice sites or transcription start sites. These partially overlapping proteins are likely to share some properties in common, and it is traditional to cluster them into “genes”. On the other hand, they are also likely to have distinct properties and roles in the functioning of the organism. For this reason, there is precedent for including all variant isoforms when defining the “proteome” [17]. Finally, it is increasingly clear that the transcribed regions of the genome form a complex web of overlaps, casting serious doubt on the utility of the gene concept [1,13].

### Length Distribution and Artefacts

The 31,035 proteins have a smooth length distribution that peaks between 100 and 200 aa (Figure 1D). Twelve percent of these proteins, 3,701, are under 100 aa: a 3-fold greater fraction than suggested by the protein collections in Figure 1A–1C. Some of these short proteins are clearly artefacts: for instance, the shortest consists of a single amino acid (methionine). In this case, CRITICA initially identifies a larger region of protein-coding sequence, but fails to find the true start codon, presumably because of some cloning or sequencing error. Unfortunately, it is extremely difficult in general to determine whether cDNAs are real or artefactual. In the following, we consider likely artefacts that might cause a bias in the length distribution, and using conservative criteria we show that they do not have a large effect on the proportion of proteins shorter than 100 aa. These results are summarised in Table 2 and detailed in Dataset S3. We have updated the CDS annotations in DDBJ/EMBL/GenBank for 521 ORFs less than 100 aa that pass all of these criteria and differ from the original annotations.

**Table 2:**
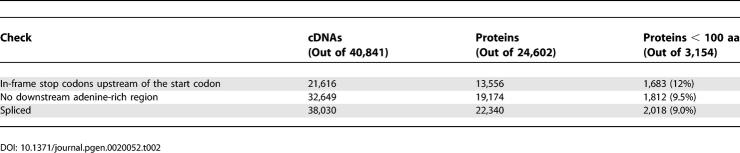
Checks of the Genome-Confirmed CDS Predictions

### Sequencing Errors

The protein predictions are sensitive to sequencing errors, especially any that introduce frameshifts into the coding region. We might expect such errors to inflate the number of short proteins, since stop codons should be common in non-coding frames. To rule out sequencing errors, the genomic sequence corresponding to each cDNA was used to confirm the ORFs. In 8,108 cases the ORF was not confirmed for various reasons (Table 3), indicating errors in the cDNA sequence, or in the draft genome sequence, or failure of the cDNA–genome mapping. The remaining 40,841 ORFs, encoding 24,602 proteins, are very unlikely to be sequence error artefacts since they are confirmed by independent cDNA and genomic sequences. The confirmed proteins have a slightly increased fraction, 13% (3,154), shorter than 100 aa. This increase can be explained because long ORFs are more likely to contain errors and get removed by our procedure. In any case, sequencing error does not have a large effect on the length distribution.

**Table 3:**
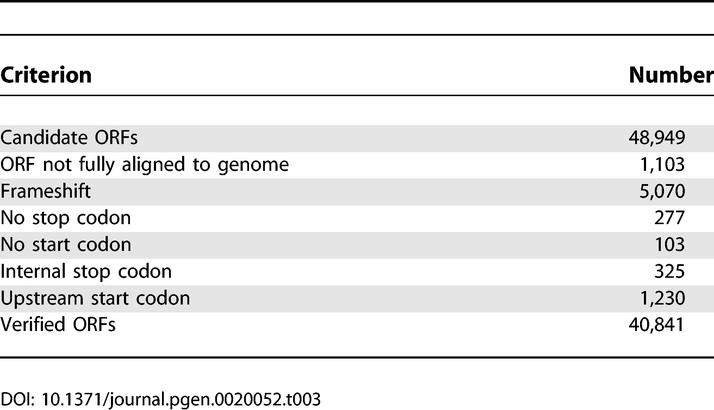
Genome-Based ORF Verification

### cDNA 5′ End Truncation

The FANTOM cDNAs were obtained using cap-trapping and other techniques to ensure that they extend all the way to the capped 5′ ends of transcripts [18]; nevertheless, we cannot rule out that some may be truncated. A large truncation might cause the cDNA to start within the coding region, in which case our procedure will predict a shortened ORF using a downstream ATG. Thus, 5′ truncations may cause a bias towards short proteins. (On the other hand, 3′ truncations would not result in protein predictions since they lack stop codons.) Since it is hard to rule out 5′ truncation of cDNAs, we tackled this problem by checking for in-frame stop codons upstream of the start codon. These are present for 21,616 out of 40,841 genome-confirmed ORFs, guaranteeing that any truncations of these cDNAs do not bite into the coding regions. This test is conservative, and likely many of the remaining cDNAs are also full-length. The 21,616 cDNAs encode 13,556 proteins, including 1,683 (12%) shorter than 100 aa. Thus, the proportion of proteins under 100 aa does not change when considering a conservative full-length subset.

### Intron Retention

Another possible artefact is that cDNAs may be primed from adenine-rich tracts in unspliced pre-mRNA or genomic DNA, and include intronic as well as exonic sequences. Such cDNAs could result in short protein predictions around single exons, and so this phenomenon could skew the protein length distribution. Two conservative measures were taken to address this problem: eliminating cDNAs that map adjacent to adenine-rich tracts on the genome and eliminating unspliced cDNAs. The FANTOM cDNAs have a bimodal distribution of adenine richness in the immediate downstream genomic sequence, with about a third (33,952) in an adenine-rich mode (more than 10 adenines in 20 nt) that is not observed for random genomic locations. This mode might correspond to internal priming, where the oligo-dT probes used in cDNA construction hybridise to adenine-rich stretches within transcripts rather than to the poly-adenine tails. On the other hand, many adenine-rich tracts in the genome come from retrotransposed poly-adenine tails, which would possess 3′-end formation signals, so we might expect many transcripts to end at these locations. In any case, removing the cDNAs adjacent to adenine-rich regions leaves 32,649 out of 40,841 genome-confirmed ORFs encoding 19,174 proteins, including 1,812 (9.5%) shorter than 100 aa. Unspliced cDNAs may come from pre-mRNA with retained introns, or mature but intronless transcripts. Removing unspliced cDNAs leaves 38,030 out of 40,841 genome-confirmed ORFs encoding 22,340 proteins, of which 2,018 (9.0%) are shorter than 100 aa. These results suggest that intron retention artefacts may indeed cause a slight bias towards short proteins. On the other hand, some genes are genuinely intronless, and it is plausible that they disproportionately encode short proteins (if long proteins require longer mRNA and longer mRNA is more likely to be spliced).

### Length Bias in CRITICA?

Since coding ORFs provide comparative and statistical evidence across their lengths, long ORFs tend to have more evidence than short ORFs, which may bias CRITICA's predictions against short proteins. In particular, it has been argued that sequence statistics as used in CRITICA's second phase are inadequate to discriminate short proteins [7]. So the CRITICA predictions might include a length-biased subset that is based on non-comparative evidence only. To rule out this possibility, we firstly note that MEGABLAST alignments with 97% or less identity were found for 100,982 out of 102,801 cDNAs, so there are hardly any predictions with no comparative evidence at all. (CRITICA does not use alignments more than 97% identical because they are uninformative for the substitution analysis.) Secondly, we re-derived the protein predictions using the results of CRITICA's first, comparative-only iteration. In this case, 29,529 proteins are predicted in 46,520 cDNAs, including 2,892 (9.8%) shorter than 100 aa. So the fraction of short proteins is actually a bit lower than in the full CRITICA results. This can be explained by the non-comparative results adding evidence to borderline cases, which tend to be short, to bring them below the *p*-value threshold.

**Figure 2:**
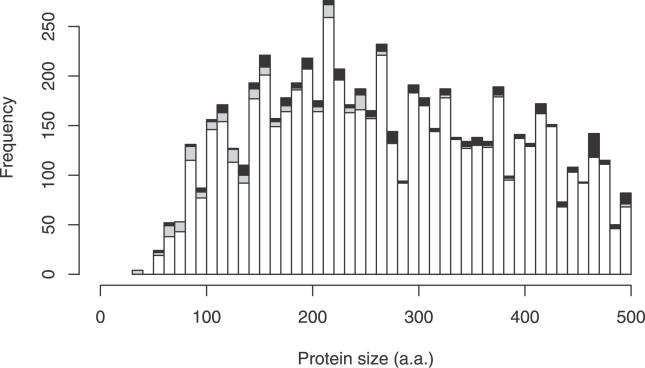
Slight Length Dependence of CRITICA Predictions Black bars indicate all mouse Swiss-Prot proteins in FANTOM. Grey bars indicate the subset of these that use the most upstream possible start codon. White bars indicate the subset of the mouse Swiss-Prot proteins in FANTOM that are predicted by CRITICA.

In order to test further whether CRITICA exhibits any length bias, we examined which known mouse proteins from Swiss-Prot it identifies. Swiss-Prot mouse proteins were aligned to the FANTOM cDNAs using BLASTX, and 8,113 cDNAs that aligned to a protein across its entire length without any mismatches or gaps were retained as the query set. In 334 cases the protein does not begin at the upstream-most ATG, and so our CRITICA pipeline has no chance of identifying the protein exactly. However, in 7,580 cases CRITICA identifies the protein in perfect agreement with Swiss-Prot. CRITICA successfully identifies both long and short proteins (Figure 2). It misses the shortest protein (sarcolipin [SARCO_MOUSE], 31 aa), but it finds the second shortest one (cardiac phospholamban [PPLA_MOUSE], 52 aa). Cases that do not begin at the upstream-most ATG are randomly distributed across different protein lengths (black bars in Figure 2). The remaining cases where CRITICA fails are concentrated at short lengths (grey bars in Figure 2). This observation must stem at least partly from the fact that Swiss-Prot does contain some errors, and that dubious Swiss-Prot entries tend to be short [19]. This result is also consistent with an expected slight bias of CRITICA against identifying short proteins, but the important conclusion is that it successfully identifies nearly all proteins down to a size of 50 aa, if not smaller.

### CRITICA False Positives?

We have shown that CRITICA finds protein-coding regions accurately when there is one to find, but it remains possible that the program falsely predicts short proteins in non-protein-coding transcripts. To assess this possibility, CRITICA was applied to transcripts from 112 mouse and human non-coding genes gathered from the literature (Dataset S4) [20]. (Since CRITICA's hexamer analysis requires that some coding regions be present, these sequences were supplemented with mouse and human protein-coding sequences from Swiss-Prot.) The program makes coding predictions for just five non-coding genes, suggesting a 4% false positive rate (Table 4). Furthermore, only two of these predictions are less than 100 aa.

**Table 4:**
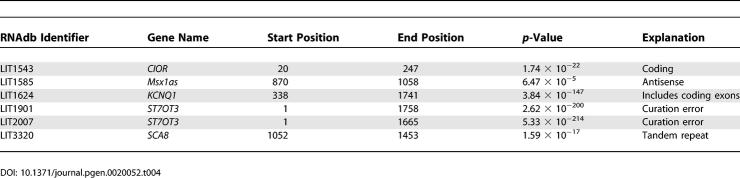
CRITICA Predictions in Curated Non-Protein-Coding RNA Sequences

Since a 4% false positive rate would amount to a significant number in the context of the ~30,000 non-coding RNAs in FANTOM, we examined these five cases in detail to understand why they are predicted to be coding. *ST7OT3* is a non-coding gene that overlaps with a coding transcript *(ST7)* [21]; the sequences represented by LIT1901 and LIT2007 were previously annotated as *ST7OT3* in GenBank; however, this annotation has subsequently been changed to *ST7,* so the inclusion of these sequences here is a curation error. *KCNQ1* is also a protein-coding gene with an isoform (LIT1624) that is probably untranslated since it introduces a premature stop codon in the second exon [22]. Since this sequence includes exons that are translated in other contexts, CRITICA unsurprisingly detects a strong coding signal. In our characterisation of the FANTOM CRITICA predictions below, we discriminate between those that have longer isoforms, some of which might actually be non-coding in the fashion of LIT1624, and those that do not. The predicted coding region in *SCA8* is mostly a triplet repeat encoding 93 leucines [23]: CRITICA's *p*-value calculation assumes that neighbouring nucleotides evolve independently, so it may be unreliable for repetitive sequences. This issue does not affect many FANTOM sequences: only 425 out of 31,035 predicted proteins contain homopolymer tracts 10 aa or longer in length, including just 16 out of 3,701 proteins less than 100 aa. Despite the original authors being unable to detect an in vitro translation product from *CIOR* [24], bioinformatic evidence including 82% identity to a hypothetical protein from *Xenopus* (AAH64217) by BLAST and a favourable context for translation initiation strongly suggests that *CIOR* encodes a short peptide. *Msx1as* is an antisense transcript of *Msx1/Hox7* [25], and since CDSs tend to mutate at every third “wobble” base-pair, there is also a coding-like 3-bp periodicity of mutations on the antisense strand [15]. CRITICA attempts to suppress antisense predictions, but it may not always succeed, especially if the sense-strand ORF is incomplete. This problem also affects few FANTOM predictions: only 283 out of 31,035 predicted proteins are in antisense relationships with one another, including just 37 out of 3,701 proteins less than 100 aa. Thus, all five cases can be explained as either curation errors or special cases (antisense, repetitive, or isoforms of coding genes) that can be flagged a priori.

### RNA Size Bias?

**Figure 3:**
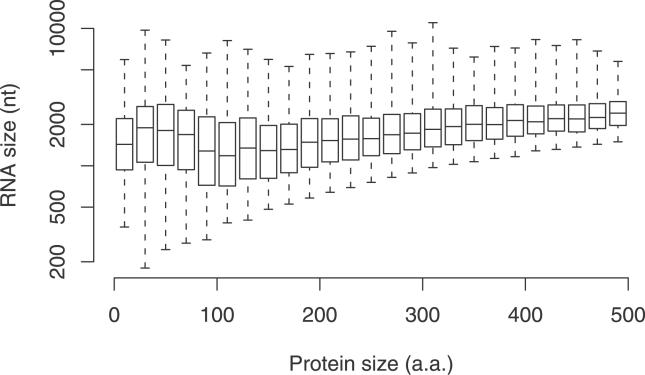
Box-and-Whisker Plots of RNA Sizes for Different Ranges of Protein Size The centre lines indicate the medians, the top and bottom of the boxes indicate the first and third quartiles, and the whiskers extend to the most extreme data points.

FANTOM, in common with most other cDNA collections, excludes transcripts shorter than about 500 nt. Since proteins less than 100 aa could be (though need not be) encoded in transcripts of size 300 nt or shorter, this might introduce a bias against finding short proteins. To assess this possibility, we examined the range of RNA lengths that encode proteins of different sizes (Figure 3). For each protein size range, the centre of the RNA length distribution remains comfortably above 500 nt. While we cannot rule out a distinct population of RNAs less than 500 nt that encode short proteins, these results suggest that FANTOM cloning constraints do not exclude most short proteins.

### Short Protein Characterisation

These results suggest that proteins less than 100 aa constitute a 3-fold greater fraction of the proteome than previously estimated. So what are all these new short proteins? Most of them are actually splice variants of longer proteins: genomic overlaps among the 31,035 predicted proteins reveal that 2,354 out of 3,701 proteins less than 100 aa are variants of proteins greater than 100 aa. This splice-variant subset will include most of the artefacts, since truncated and immature cDNAs will overlap their full-length counterparts. Eliminating all proteins that overlap a longer variant on the genome leaves 16,900 maximal-length isoforms, of which 1,240 (7.3%) are less than 100 aa.

The vast majority of proteins less than 100 aa are absent from previous catalogues of mouse proteins: only 232 out of 3,701 short proteins match entries in the mouse IPI database (BLASTP alignment with 95% or greater identity and covering 95% or greater of both sequences). Furthermore, 495 out of 3,701 short proteins lack similarity to any known protein, based on searching the UniRef90 database. These proteins that are both short and dissimilar to any known protein are invisible to most protein identification methods and merit the label “dark matter”.

### Overlap with Gene Predictions

**Figure 4:**
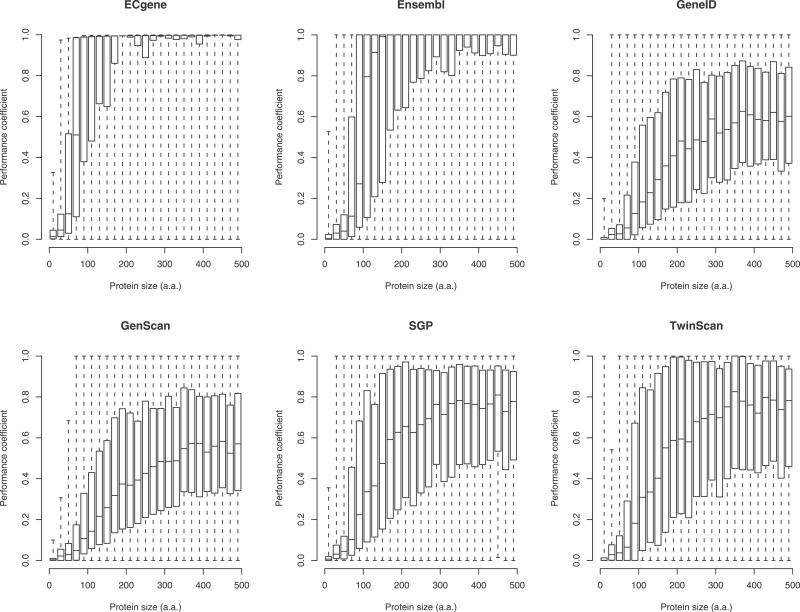
Overlap of FANTOM CRITICA Predictions with Genome-Based Gene Predictions Made by Six Methods Only the 16,900 maximal-length isoforms of the FANTOM CRITICA predictions were considered; these were compared to each genome-based method in turn as follows. Each CRITICA prediction was compared to the genome-based gene prediction that overlapped it by the greatest number of nucleotides, and the degree of overlap was quantified using the performance coefficient: the number of nucleotides in the intersection of the two predictions divided by the number of nucleotides in the union of the predictions [45]. These are box-and-whisker plots: the centre lines indicate the medians, the top and bottom of the boxes indicate the first and third quartiles, and the whiskers extend to the most extreme data points.

As expected, the 16,900 maximal-length isoforms show significant overlap with genome-based gene predictions, but the overlap falls off rapidly below 100 aa (Figure 4). GenScan and GeneID are ab initio gene predictors [26,27]: they rarely agree perfectly with CRITICA even for proteins greater than 100 aa, although there is usually significant overlap. It is possible that they identify different splice variants. For proteins less than 100 aa the overlap tends to be very low, but usually greater than zero. TwinScan and SGP are variants of GenScan and GeneID, respectively, that incorporate comparative analysis not unlike that used by CRITICA, although they tackle a much harder problem since they are applied to the genome sequence and must predict splicing patterns [28,29]. TwinScan and SGP show significantly greater overlap with CRITICA than GenScan and GeneID for proteins greater than 100 aa, but not for proteins less than 100 aa. Ensembl and ECgene use empirical data such as expressed sequence tags (ESTs) to assist gene prediction [30,31]; for proteins greater than 100 aa they tend to agree perfectly with the CRITICA maximal-length isoforms, but there is still a marked drop-off below 100 aa. ECgene agrees significantly better with CRITICA than does Ensembl, showing good agreement even for proteins in the range 80–100 aa. This must partly be because ECgene makes about an order of magnitude more predictions than the other methods, including splice variants.

### Protein Domains and Motifs

Recognisable functional domains were found in only 384 out of 1,240 maximal-length isoforms less than 100 aa, based on searching the Pfam database [32]. Some functional categories of domains are significantly overrepresented among short proteins (Table 5). However, these categories largely reflect previously known roles of short proteins. These results suggest that targeted functional studies will be needed to elucidate the functions of most novel short proteins.

**Table 5:**
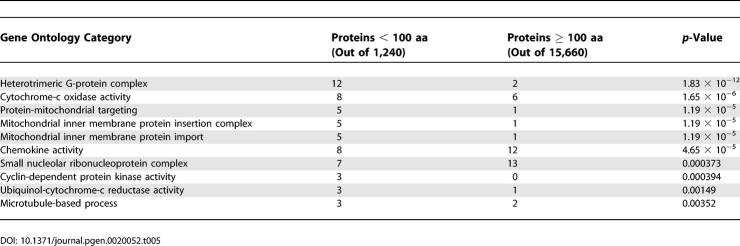
Ten Most Overrepresented Gene Ontology Categories for Pfam Domains in Proteins Less Than 100 aa

We also provide SignalP and TMHMM predictions for all small ORFs in Datasets S5 and S6. SignalP predicts signal peptides in 125 out of 1,240 small ORFs. The reported false positive rate for SignalP [33] is 15%, but that is based on a test set containing a higher proportion of signal peptides (34.5%) than is predicted in the overall mouse genome (20%). Using an adjusted false positive rate of 27% for SignalP predictions on the mouse genome, we predict that approximately 91 small ORFs contain signal peptides. It is not surprising that there is a substantially lower fraction of signal peptides in the overall mouse genome (7.3% versus 20%) since cleaved proteins resulting from signal-peptide-containing small ORFs are extremely small, limiting their usefulness and scope.

### Paralogue Clusters

Although most of the short proteins do not harbour known domains, it may be possible to detect novel domains by clustering together proteins with similar sequences. Using BLASTCLUST, 117 out of 1,240 short proteins were grouped into 38 families with two or more members (Dataset S7). Many of these families share a Pfam domain in common; for instance, the 20 members of the largest family each possess a KRAB domain (Krüppel-associated box). There are also a few novel domains; for instance, the proteins encoded by clones 1500011E09, 6330419J24, and F430011G22 share a ~45-aa segment with ~75% identity that does not match any Pfam domain.

### Amino Acid Composition

Short proteins contain slightly different proportions of amino acids from long proteins (Table 6). The most marked difference is an excess of cysteine in short proteins, possibly indicating that they more frequently use disulphide bonds to maintain their structures. Short proteins also have an excess of arginine and glycine, and a dearth of aspartate, histidine, asparagine, and threonine. Short mouse proteins from Swiss-Prot generally have the same biases as the FANTOM CRITICA proteins, except that the differences from long proteins are more extreme.

**Table 6:**
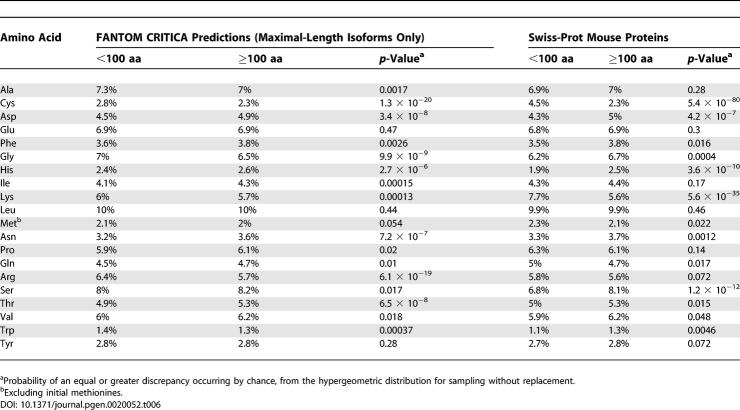
Amino Acid Composition of Short and Long Proteins

### Evolutionary Conservation

**Figure 5:**
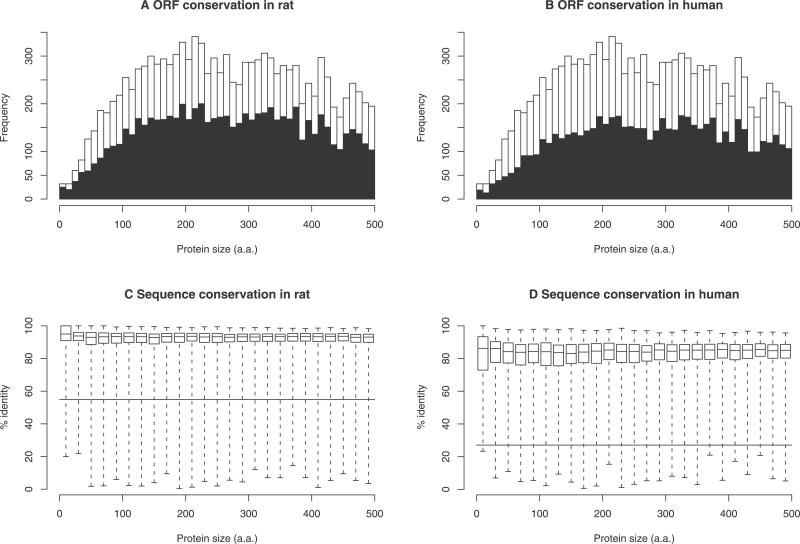
Evolutionary Conservation of FANTOM CRITICA Predictions Only the 16,900 maximal-length isoforms of the FANTOM CRITICA predictions were considered. (A) Histogram of predictions where the reading frame is perfectly conserved in rat (black) or disrupted (white). (B) Histogram of predictions where the reading frame is perfectly conserved in human (black) or disrupted (white). (C and D) Sequence conservation of predictions versus (C) rat and (D) human. Sequence conservation was quantified by the percentage of nucleotides in each predicted protein-coding region that align to identical nucleotides in the other organism. These are box-and-whisker plots: the centre lines indicate the medians, the top and bottom of the boxes indicate the first and third quartiles, and the whiskers extend to the most extreme data points. The long horizontal lines indicate the percentage of sequenced nucleotides in the mouse genome that align to identical nucleotides in the other organism.

In order to characterise the short proteins further and obtain more evidence regarding their coding status, we investigated their degree of conservation in rat and human. We extracted rat and human sequences aligned to the predicted coding exons from the University of California Santa Cruz BLASTZ net whole-genome alignments [34]. Firstly, we checked conservation of the reading frame, requiring that the rat or human sequences form a translatable ORF (length divisible by three, beginning with a start codon, ending with a stop codon, and having no intervening stops) and that they cover the whole CRITICA-predicted ORF from start to end (but allowing gaps in the middle). About two-thirds of the 16,900 maximal-length isoforms are conserved in rat and about half in human (Figure 5A and 5B). The non-conserved cases often have minor disruptions, such as a frameshift near the end of the ORF that causes a different stop codon to be used. The proportion of conserved ORFs is roughly the same for long and short ORFs. Secondly, we examined sequence conservation, by counting the percentage of nucleotides in CRITICA ORFs that align to identical nucleotides in the other species. As expected, these sequences are much more conserved than the genome average (Figure 5C and 5D). Short ORFs are conserved equally well as long ORFs, providing further evidence for their protein-coding status.

### Transcriptional Support for Small ORF Transcripts

Firstly, in order to provide transcriptional support for the validity of small-ORF-encoding clones, the number of independent ESTs for each transcript in all public data was calculated. In total, 1,167 of the 1,240 small-ORF RNAs were supported with at least one other RNA or EST sequence from independent cDNA clones, based on genomic mappings. The median number of supporting ESTs and cDNAs for the set was 26, suggesting that the small-ORF cDNAs described in this study are generally derived from well represented transcripts.

**Figure 6:**
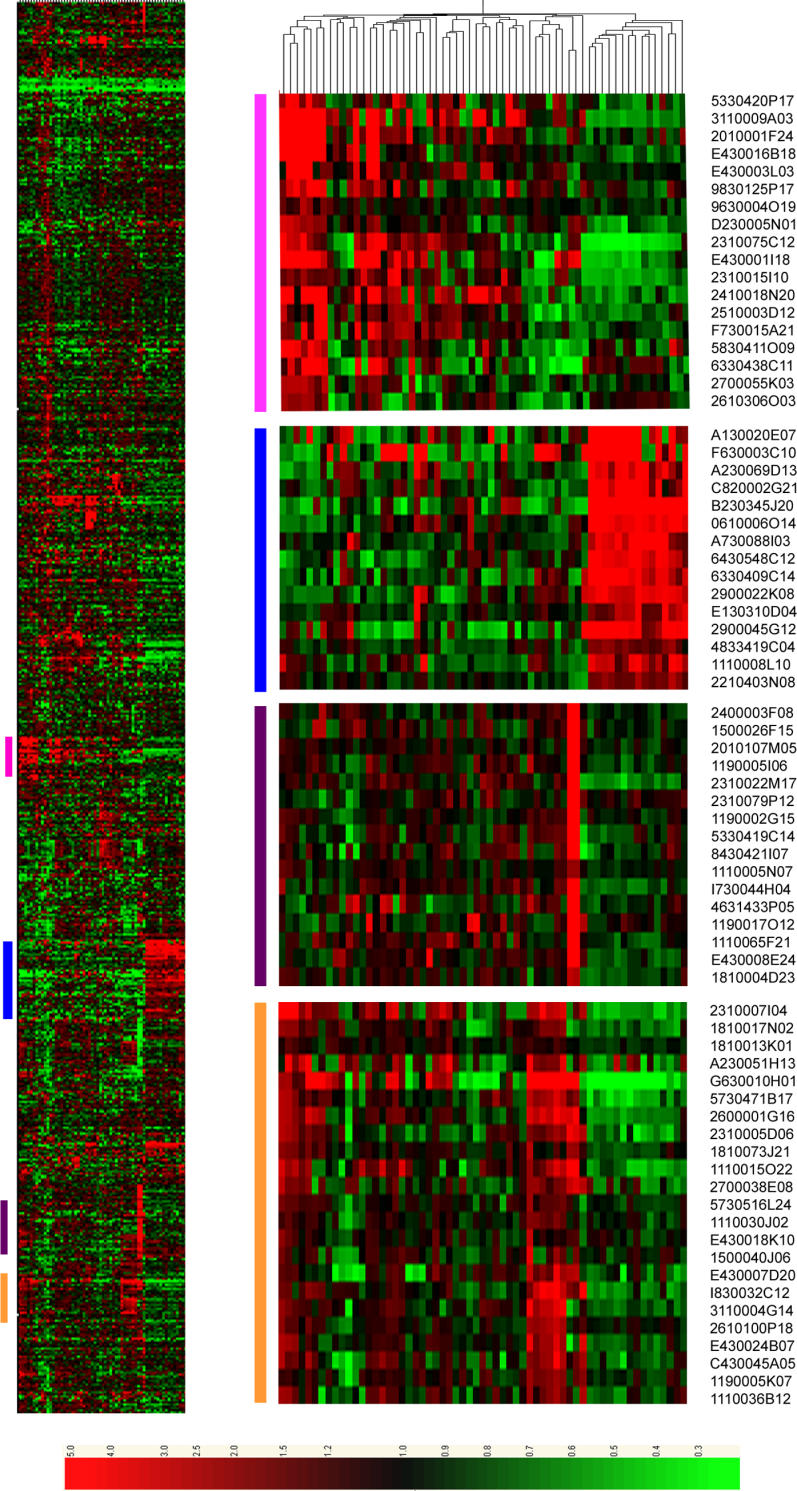
Heat Map Displaying Relative Expression Levels of Small-ORF Transcripts Present within 61 Mouse Tissues from the Genomics Institute of the Novartis Research Foundation GeneAtlas Small-ORF transcripts are clustered on the vertical axis, and tissue samples are along the horizontal axis. All gene expression is displayed relative to the median level of each transcript across all 61 tissues. The coloured columns on the left-hand side of the heat map (left) correspond to the blown up sections (right). FANTOM3 clone identifiers are included in the blown up clusters. A blow-up of the tissue clustering, including the tissue names, is available as Figure S1.

Secondly, we also reviewed gene expression patterns of small-ORF-encoding RNAs within the mouse Symatlas 61 tissue atlas Affymetrix dataset [35] to provide functional significance for these largely uncharacterised transcripts. There were 844 small-ORF transcripts specifically identified within this dataset. The mean expression level of the small-ORF transcripts did not differ significantly from that observed for known coding transcripts (data not shown). Hierarchical clustering of the data for these 844 transcripts revealed that the majority of them are expressed in a highly tissue-restricted fashion (Figure 6). Several large clusters of small-ORF transcripts were observed. The largest clusters included neural tissues (15 sub-dissected regions of the brain plus the pituitary), haemopoietic cells and tissues (T cells, B cells, lymphocytes, spleen, and bone marrow), and embryonic cells and tissues (whole-embryo 6.5, 7.5, 8.5, 9.5, and 10.5 days post-copulation and unfertilised egg). The highly restricted expression patterns of these transcripts further support the legitimacy of these transcripts and suggest a functional role in specific tissues.

### Translation and Localisation Assays

Finally, we attempted to provide supporting evidence for a subset of short ORFs identified by CRITICA by the transient expression of tagged candidate cDNAs in mammalian cells. A panel of mammalian expression plasmids was generated that contained the small ORF and its native 5′ UTR fused to the ORF encoding green fluorescent protein (GFP). The 5′ UTR was retained for each small ORF in order to test the strength and legitimacy of its initiation codon. The GFP ORF was also designed to lack an initiation codon so that GFP protein could be observed only if the candidate small ORF was efficiently translated.

**Figure 7:**
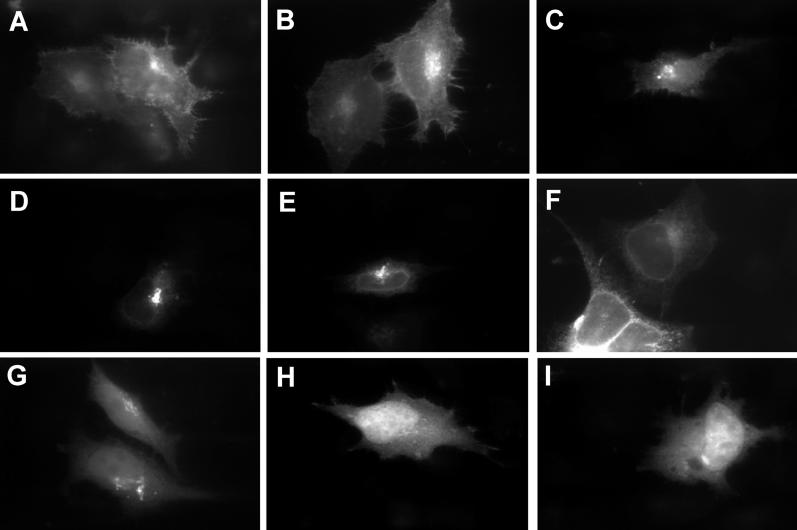
Observed Subcellular Localisations for Small SignalP Positive ORFs Predicted by CRITICA, Fused to GFP (A–C) Cell surface and peri-nuclear localisations of A430023G14, 1110065P19, and 5430416O09. (D and E) Nuclear envelope and peri-nuclear golgi-like localisations of E030042M04 and 1500009C09. (F) Endoplasmic-reticulum-like staining of C230071E12. (G) Peri-nuclear staining of 0610011H04. (H and I) GFP-like ubiquitous staining of D330006H24 and A630083C19, similar to that observed for 1700084P19, D630042J06, F730009G16, 5430411J08, and D130012G24.

A total of 25 small ORFs were selected on the basis of having strong transcriptional support based on EST counts and expression profiling data from the Genomics Institute of the Novartis Research Foundation (Table S1). Upon transient transfection into HeLa cells, 14 of these cDNAs, including seven dark matter cases, generated GFP-tagged proteins via immuno-fluorescence (Figure 7). Three of the tested ORFs localised to the cell surface and gave endoplasmic-reticulum-like staining (5430416O09, A430023G14, and 1110065P19); the remaining four gave peri-nuclear, endoplasmic reticulum/golgi-like localisations (0610011H04, 1500009C09, C230071E12, and E030042M04). All of these small ORFs were predicted to possess either trans-membrane domains via TMHMM [36] or signal peptide motifs via SignalP [33], suggesting correct targeting. Another seven candidate transcripts generated GFP-tagged protein that was observed throughout the cell. This success rate for detecting expression of tagged constructs in a single cell type compares favourably to rates observed for other screens such as the human ORFeome (55% overall success by immuno-fluorescence or Western blotting for 282 constructs) [37]. These data support that small ORFs identified via this computational screen are capable of generating efficiently translated peptides.

## Discussion

This study provides the first reasonably accurate picture of the mammalian short proteome. Although CRITICA reveals three times more short proteins than previously thought, it nevertheless shows that protein abundance peaks between 100 and 200 aa, and declines fairly rapidly below 100 aa. It seems to be a lucky coincidence that the turning point occurs close to the traditional 100-aa threshold used for protein identification. This distribution may tell us something about the minimal size of a useful protein: it may be difficult to encode a functional domain plus necessary localisation peptides in much less than 100 aa. This finding also suggests that short proteins may perform atypical functions.

Importantly, the comparative approach is able to identify dark matter that would be invisible to most standard protein identification methods. The GFP assays demonstrate that some of these novel proteins can be translated in the context of their native 5′ UTR and undergo localisation to specific subcellular compartments, further evidence that they are real and functional. It is likely that these proteins perform very novel types of biological function.

Production of a precise catalogue of short proteins awaits an accurate way to exclude cDNA artefacts. This is a hard problem because even if we see a retained intron that splits a protein-coding region, or a cDNA that starts in the middle of an ORF, we cannot tell whether these are artefacts or genuine variant transcripts. Recent CAGE and RACE data suggest that many cDNAs that would previously have been annotated as truncated are in fact full-length transcripts from alternative promoters [1].

Recently, gene prediction in mammalian genomes has improved through the use of comparative methods [38]. Our work highlights the benefits of marrying this approach more closely with transcript data. Use of transcripts solves several issues that are largely intractable for current genome-based predictors, such as identification of multiple splice variants, overlapping or embedded genes, giant introns, and non-canonical splice sites. We imagine a hybrid gene prediction method that uses potentially artefactual cDNA and EST data to guide but not limit predictions in the genome sequence.

CRITICA's predictions are quite reliable in the range of 50–100 aa, but the reliability below 50 aa remains uncertain. There must come a point when the ORF is too short for CRITICA to have any chance of detecting a statistically significant signal, and so the extremely short peptidome remains hidden. It is also possible that tiny proteins are encoded in tiny RNAs, which are excluded from most transcript datasets. Furthermore, this study has ignored upstream ORFs, which may contribute many short proteins [39]. We cannot rule out that there are thousands of very short proteins, say less than 10 aa.

## Materials and Methods

### Protein size data.

FANTOM full-length protein annotations in mature cDNAs were obtained from ftp://fantom.gsc.riken.jp/fantomdb/3.0/anndata.txt.gz. Identical proteins were merged as described below. Mouse IPI proteins were taken from IPI version 1.22. Human Swiss-Prot proteins were taken from the human.seq file downloaded on 4 March 2005; proteins whose description ends in “(Fragment)” or “(Fragments)” were removed.

### CRITICA.

The CRITICA pipeline was designed for bacterial genomes and the smaller sequence databases of 1999. Some modifications were necessary to cope with the 200-million-nucleotide FANTOM dataset and the 10-billion-nucleotide nonredundant GenBank database. Firstly, to prevent excessive database hits, we worked with RepeatMasked FANTOM sequences obtained from ftp://fantom.gsc.riken.jp/FANTOM3/repeats/fantom3_total103k_r2.masked.fasta.gz. The FANTOM sequences were aligned to National Center for Biotechnology Information's nt database (January 2004) using MEGABLAST with options –e (1e − 4) –D 1 –F “m D” –U T –J F –f T –t 18 –W 11 –A 50 –q −2 –G 5 –E 2 [40]. These options cause MEGABLAST to use discontiguous seeds that allow mismatches every third position, which should be more sensitive for finding homologues of protein-coding regions. CRITICA version 1.05b was modified to accept large files, and the alignments were analysed with the options –iterate-critica –no-sdscores –fraction-coding = 0.5 –genetic-code = 1 –frameshift-threshold = 10.

The non-coding RNAs (Dataset S4) were analysed in the same way after processing with RepeatMasker open-3.0.8 with options –xsmall –s (and –species mouse for the mouse sequences) [41].

### Redundancy elimination.

The ORF–cDNA annotations were composed with the cDNA–genome mappings provided by the FANTOM3 consortium (ftp://fantom.gsc.riken.jp/FANTOM3/mapping_materials/f3_mm5_best.psl.gz) to obtain ORF–genome alignments. Unmapped ORFs were discarded, but partially mapped ORFs were retained. ORFs with identical lengths and genome alignments were grouped, and one representative was arbitrarily chosen from each group.

### Genome-based ORF verification.

FANTOM cDNA–genome mappings with exon/intron definitions were obtained from ftp://fantom.gsc.riken.jp/FANTOM3/mapping_materials/f3_mm5_best.gff.gz. These mappings were used to construct “virtual cDNAs” by concatenating the genomic exons aligned to a cDNA (M. Furuno, unpublished data). cDNA–virtual cDNA alignments were constructed based on non-intron gaps in the cDNA–genome mappings. The ORF–cDNA annotations were then composed with the cDNA–virtual cDNA alignments to obtain ORF–virtual cDNA alignments (“virtual ORFs”). Alignments not reaching both ends of the ORF were discarded. Finally, we verified that the virtual ORFs were in fact maximal ORFs, with length divisible by three, bounded by start and stop codons, lacking internal stop codons, and without alternative start codons further upstream.

### Splicing criteria.

A cDNA was considered spliced if it had multiple exons according to the FANTOM cDNA–genome mappings and, moreover, had an intron flanked by GT-AG with alignment gaps in the cDNA only.

### Protein database searches.

We used NCBI BLAST 2.2.9 [42] for protein searches. The FANTOM cDNAs were aligned to mouse Swiss-Prot proteins (mgd.seq downloaded 1 April 2005) using BLASTX with options –m 8 –F “m S” –S 1 –g F –e 0.01 –M PAM30 –y 1. BLASTP was used to find database matches for predicted proteins. The mouse IPI database was searched without low-complexity masking, since we were looking for almost exact matches. The UniRef90 database (downloaded 9 January 2004) was searched with an *E*-value threshold of 0.01 [5].

### Gene predictions.

Gene predictions were downloaded from http://hgdownload.cse.ucsc.edu/goldenPath/mm5/database on 14 October 2005.

### Pfam search.

The predicted proteins were searched against Pfam version 17 using pfam_scan.pl version 0.5 (http://www.sanger.ac.uk/Users/sgj/code/pfam/scripts/search/pfam_scan.pl) and HMMER 2.3.2 (http://hmmer.wustl.edu).

### Paralogue clusters.

To determine paralogue clusters, BLASTCLUST 2.2.11 was used with options –S 0.0 –L 0.0, after filtering low-complexity sequences with SEG [43].

### Conservation analysis.

The FANTOM cDNA–genome alignments (f3_mm5_best.gff) were composed with whole-genome alignments, obtained from http://hgdownload.cse.ucsc.edu/goldenPath/mm5/vsRn3/axtNet and http://hgdownload.cse.ucsc.edu/goldenPath/mm5/vsHg17/axtNet, to obtain alignments of the CRITICA ORF predictions to the rat and human genomes. Alignments inconsistent with *cis*-splicing (e.g., involving multiple chromosomes) were discarded.

### Expression profiling.

Normalised microarray expression data for small-ORF transcripts were taken from the large-scale mouse transcriptome analysis of Su et al. [35] (GEO accession GDS592). Probesets that were diagnostic for small-ORF transcripts were identified by BLAST mapping. These data were hierarchically clustered using Genespring version 7.2 as described previously [44].

### Translation and localisation assays.

Gene-specific primers were designed to the C-terminal sequences of 25 short ORFs identified by CRITICA and also predicted to contain signal peptides (SignalP) (Table S2). These primers were then used in conjunction with a vector-specific primer to amplify a region comprising the 5′ UTR and ORF of these identified short ORFs from FANTOM full-length cDNA clones. Using a fusion PCR consisting of the primary PCR product, a CMV promoter fragment, and a GFP-SV40 terminator fragment, a full-length linear expression construct was generated. This full-length product consisted of—from 5′ to 3′—a CMV promoter, the 5′ UTR and short ORF of the clone to be tested, and a GFP tag fused in-frame at the C-terminus of the ORF followed by two copies of the SV40 terminator sequence (Figure S2).

The linear expression constructs generated by fusion PCR were transiently transfected into HeLa cells. Transfections were carried out for 16 h using Effectene (Qiagen; http://www.qiagen.com) in 24-well plates using cells grown on coverslips. Cells were fixed in 4% paraformaldehyde, and subcellular localisation of fusion proteins was visualised at 100× magnification by GFP fluorescence.

## Supporting Information

Dataset S1 CRITICA CDS Predictions in the FANTOM cDNA Set This dataset is in GFF3 format, as described at http://song.sourceforge.net/gff3.shtml.
(2.2 MB DOC)

Dataset S2 Protein Sequences Predicted by CRITICA in the FANTOM cDNA Set (FASTA Format) (6.0 MB ZIP)

Dataset S3 Checks of the FANTOM CRITICA CDS Predictions (908 KB TXT)

Dataset S4 Curated Non-Coding and Coding Transcripts Used to Test CRITICA's Specificity (FASTA Format) (4.4 MB TXT)

Dataset S5 SignalP Predictions for Protein Sequences Predicted by CRITICA in the FANTOM3 cDNA Set (5.6 MB TXT)

Dataset S6 TMHMM Predictions for Protein Sequences Predicted by CRITICA in the FANTOM3 cDNA Set (3.3 MB TXT)

Dataset S7 Paralogue Clusters of FANTOM CRITICA Predictions among 1,240 Maximal-Length Isoforms Less Than 100 aa (1 KB TXT)

Figure S1 Enlarged Hierarchical Cluster Tree for Tissues in Figure 6
(2.4 MB TIF)

Figure S2 Diagram Describing Design of PCR-Based Generation of C-Terminally Tagged ORFs (60 KB TIF)

Table S1 ORFs Screened by Transient Translation Assay in HeLa Cells (17 KB XLS)

Table S2 Primer Sequences Used for Generating PCR-Mediated Expression Constructs (17 KB XLS)

## Abbreviations

CDS: coding sequence
EST: expressed sequence tag
GFP: green fluorescent protein
ORF: open reading frame

## Abbreviations

CDS: coding sequence
EST: expressed sequence tag
GFP: green fluorescent protein
ORF: open reading frame
