# Supplementary figures and images for: The Abundance of Short Proteins in the Mammalian Proteome
Source: PLoS Genet. 2006 Apr 28;2(4):e52. doi: 10.1371/journal.pgen.0020052 (PMC1449894; doi:10.1371/journal.pgen.0020052)

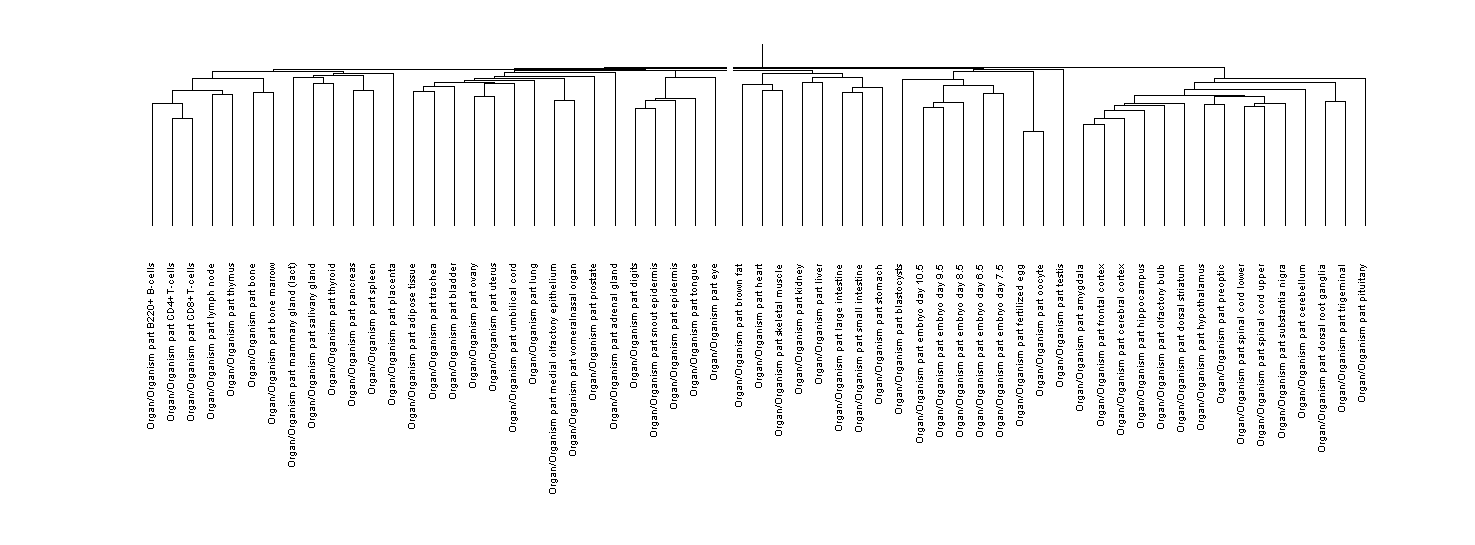

Supplement: Figure S1 — (2.4 MB TIF) [file pgen.0020052.sg001.tif]

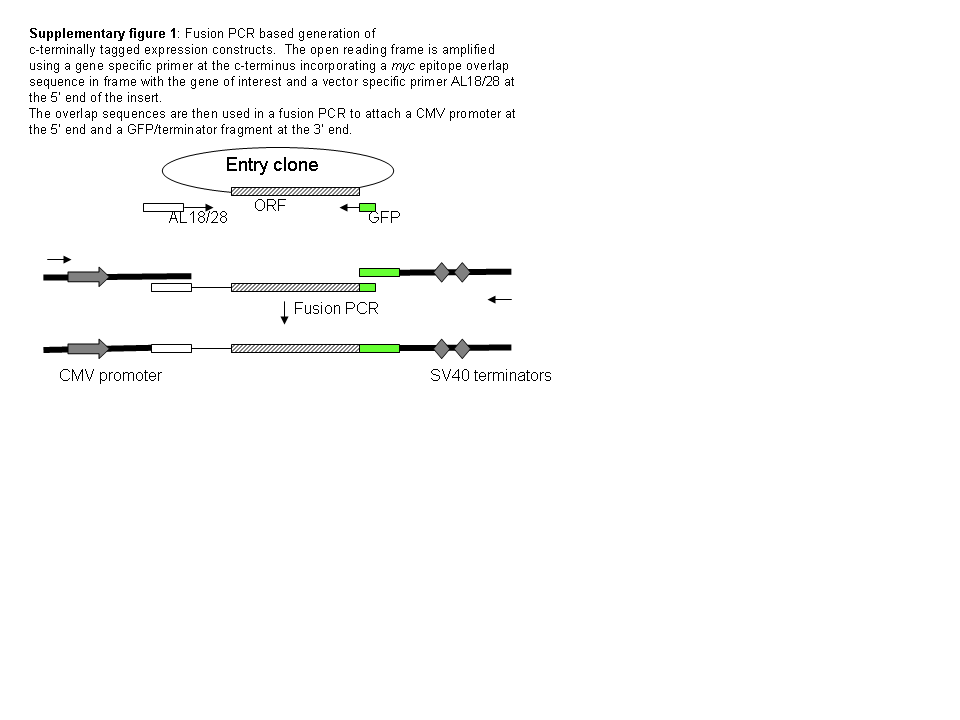

Supplement: Figure S2 — (60 KB TIF) [file pgen.0020052.sg002.tif]
